# Intelligence

**Published:** 1828-07

**Authors:** 


### INTELLIGENCE.

### MONTHLY REPORT OF PREVALENT DISEASES.

The weather, during the last month, has had but little of the appropriate characters of the season, and although at the moment we write it has become genial, yet the change is too recent to have produced any of the diseases of summer. The vicissitudes of temperature during the period comprised in the present report have produced a great number of cases of rheumatism, some of which have been rather acute. Catarrhal affections have also been prevalent, although not very severe. One of the most remarkable circumstances which has presented itself to our notice has been some cases of fever, in which the continued has passed into the intermittent form, and required the exhibition of quina. In some of these, swellings have taken place in the glands in different parts of the body, and, becoming chronic, have proved exceedingly troublesome.

---

*College of Physicians.*—A series of evening meetings have recently been held at the College of Physicians, to which all the respectable members of the profession in its different departments have been invited. These meetings have been numerously attended, and have certainly tended to produce an amicable feeling among those present. Various papers have been read, particularly one on Tic Douloureux, by Sir H. HALFORD; on Vaccination, by Dr. MACMICHAEL; on the Treatment of Inflammation, by Dr. YEATS; on Ossification of the Arch of the Aorta, by Dr. WARREN; and on some of the different forms of Mental Disease, by Dr. LATHAM.

---

Dr. HOLLAND.—We understand that this gentleman has just been added to the list of Fellows.

---

*Anatomical Committee.*—The Report of this Committee, so long expected, has not yet been presented.

---

*Westminster Hospital.*—It is in contemplation to build a new hospital, not on the site of the old one, but near St. Martin's church.—We believe the measure is not yet fully decided on.

---

*London University.*—It is announced that the Courses are to commence in October. The following are the medical arrangements:

Anatomy, every day (except Sunday), from two to half-past three,—Mr. G. S. PATTISON.

Dissections and Demonstrations, every day,—Mr. J. B. BENNETT.

Physiology, Monday, Wednesday, and Friday, from eleven to twelve,—Mr. C. BELL.

Comparative Anatomy and Zoology, Monday, Wednesday, and Friday, from three to four,—Dr. R. GRANT.

Surgery, Monday, Wednesday, and Friday evenings, from seven to eight,—(Professor not yet appointed.)

Treatment and Nature of Diseases, every morning (except Saturday), from nine to ten,—by Dr. CONOLLY.

Midwifery and Diseases of Women and Children, Monday, Tuesday, Thursday, and Friday evenings,—by Dr. D. D. DAVIS.

Materia Medica and Pharmacy, every morning (except Saturday), from eight to nine,—Dr. A. T. THOMSON.

---

*Medico-Botanical Society of London.*—A meeting of this Society was holden on Friday, the 13th instant, at its apartments, 32, Sackville-street, Piccadilly; Sir BENJAMIN HOBHOUSE, Bart. F.R.S. Vice-President, in the chair. The minutes of the last meeting having been read and confirmed, a letter from Sir JAMES M'GRIGOR, President of the Society, addressed to Mr. YOSY, Secretary, was read: it contained another from the Right Hon. ROBERT

Peel, his Majesty's principal Secretary of State for the Home Department, conveying to the Society the pleasure his Majesty felt in becoming the Patron, and expressing his best wishes for the success of their useful exertions in a very important department of science.

A letter was read from the East-India Company, informing the Society that the Court of Directors had granted them duplicates of all the medical plants in their extensive Herbarium.

A letter was read from his Majesty the King of Bavaria, announcing that the collection which his Majesty had ordered was, through the care of Professor Martius, now ready, and would be delivered to the Society in a short time by the Bavarian ambassador in London, Baron de Cetto. The collection was said to consist of upward of 600 specimens.

Mr. Frost, the Professor of Botany, then delivered a lecture on the genus Laurus, a splendid collection of which was exhibited to the members, there being no less than eighteen living species from his Majesty's gardens at Kew, furnished by the kindness of W. T. Aiton, Esq. Beside these, there were thirteen other species, contributed by Messrs. Loddige, of Hackney, Mr. Richard Forest, Mr. David Cameron, Mr. Fairburn, and Mr. Richardson. This genus is particularly rich, as it is from it that many valuable medicines are procured, such as camphor (Laurus Camphora), cinnamon (Laurus Cinnamomum), sassafras (Laurus Sassafras), bastard cinnamon (Laurus Cassiæ), &c.

A complete bowl of camphor was exhibited, as also several other pharmaceutical preparations.

The chairman had announced that a vacancy had occurred in the professorship of Materia Medica, candidates for which were requested to send in their testimonials as early as possible, as the vacancy would be filled up at the ensuing meeting.

The chairman announced that the first fasciculus of the first volume of the Transactions of the Society, illustrated with two coloured engravings of the Melaleuca Cajuputi, and Melaleuca Leucadendron, was now ready for delivery to the members.

The committee also announced, that a paper on the Doubtful Identity of Bonplandia Trifoliata and Angustura Bark, by Dr. John Hancock, would be laid before the next meeting, to be holden on the 11th July, 1828.

---

*Supply of Water to the Metropolis.*—The following remarks on this subject are made by Dr. Ryan:

Public attention has at length been aroused to the means of supplying this vast metropolis with water, a fluid so important to human existence. This has been effected by the highly meritorious exertions of an individual, (Mr. Wright, of Regent-street.) An anonymous pamphlet appeared, styled the Dolphin, a name adopted by one of the companies which supplies the western part of the town with water, which so exposed the contaminated state of the fluid supplied, that a meeting of all the rank and respectable inhabitants of that district took place, who, among other things, resolved, "That the water taken up from the river Thames at Chelsea, (the site of the Dolphin,) for the use of the inhabitants of the western portion of the metropolis, being charged with the contents of the great common sewers, (and others, perhaps 10,000 in number,) the drainings from dunghills and laystalls, the refuse of hospitals, slaughter-houses, colour, lead, and soap works, drug-mills and manufactories, and with all sorts of decomposed animal and vegetable substances, rendering the said water offensive and destructive to health, ought no longer to be taken up by any of the water companies from so foul a source;"—that the water was pronounced by professional men of the first eminence to be "a filthy fluid, loaded with decayed vegetable matter, and other substances equally deleterious to health, and unfit for domestic purposes." A petition was presented to both Houses of Parliament, praying that a commission should be appointed by his Majesty, "to inquire into the supply of water in the western part of the metropolis;" which commission was granted. The commissioners summoned a vast number of witnesses, and drew up a Report on the evidence, which extended to 154 folio pages; the substance of which was a perfect confirmation of the allegations of the petitioners, as to the impurity of the water supplied to the metropolis, but that there was an abundant supply. This Report was submitted to Parliament; but the Right Hon. R. Peel, his Majesty's Secretary of State for the Home Department, was of opinion, as the supply was abundant, that the government ought not to take up the subject, but leave it in the hands of the public. This is the most remarkable instance of the apathy or inattention of that great improver of abuses, to the public interests. Posterity will scarcely credit that a statesman, who has effected so many invaluable legal improvements, could have excluded from his consideration a subject of such vital importance to every human being in this mighty metropolis. The public at large, however, are unanimous in their decided intention of renewing their application to the legislature, which sooner or later must comply with the just and reasonable prayer of their petitions. It appears from the Report of the commissioners, that a parallel instance of gross impurity and filthiness, in the supply of water to the inhabitants of a large city, cannot be equalled in any other part of the globe. Different specimens of the water supplied—of that fluid which enters into the composition of animals and vegetables, and so powerfully and extensively assists the evolutions of the solids, and composes the greater part of the fluids in the human body,—were examined by the most eminent medical men, and analysed by our most distinguished chemists, who unanimously declared "that such specimens were loaded with a great quantity of filth, which renders such water disgusting to the senses, and improper to be employed in the preparation of food." Surely such a monstrous evil requires a remedy. That fluid, which is the vehicle of human food and nourishment, must have a constant and powerful agency on the animal machine at all times, a constant and regular operation on the human body; and hence the great necessity of the purity and salubrity of that fluid. So great has been the deterioration of the water in the Thames, that fish can no longer live in certain parts of the river; and that they cannot be preserved in such water for any length of time after removal.

The principal causes of the impure and deteriorated state of the water of the Thames, which supplies a very considerable part of the capital, are the refuse of animal and vegetable matters, which are now allowed to flow into the common sewers, and thence into the river; the refuse of the coal gas, which pollutes the river in many parts; the quantity of dead animals constantly thrown into the water; the refuse of slaughter houses; and the hideous and abominable exuviæ of one million of inhabitants. Such are the ingredients that are dissolved, mechanically suspended, or chemically combined, in the water which supplies the western, the most influential and fashionable part of the British capital.

It appears from the Report of the commissioners, that the water of the New River is by far the purest source of supply, and that it occasionally derives only a small quantity from the Thames, when there is a severe frost, or great drought. "The New River and Hampstead waters (says Dr. James Johnson) are ethereal streams, compared with those of Chelsea." The commissioners also acknowledge the superiority, but observe "there is still room for improvement." The commissioners, as if anxious to please all parties,—the public at one time and the water companies at another, (and perhaps may fail in pleasing either,)—assert that the water may be filtered in the beds of sand in the river; but, if the matters be in solution, or chemically combined, that filtration cannot be perfect, as the water can be only partially purified. They obtained specimens of the water at its average state, and after a fall of rain, and from that district where it is said to have been impregnated with copper derived from the bottoms of the ships. I have already stated the result of chemical analysis, namely, "that all the specimens being loaded with filth, which renders the water disgusting to the senses, and improper for the preparation of food;" and I may add, "that it contained no copper." Yet the able chemist, Dr. Bostock, asserts "that the greatest part, if not the whole, of the extraneous matters may be removed by filtration." The commissioners are of opinion that, if the quality of the water which supplies the capital be objectionable, any remedy of a local nature will be comparatively unimportant. On the whole, they observe that the supply is capable of, and requires improvement. They do not sanction any of the proposed plans of improvement, as their time would not allow them to investigate the subject fully; and their earnest hope is, that its full investigation, by competent persons, will not long be deferred; and that the supply ought not to be left to the discretion of the companies, and that their proceedings should be subjected to some effective superintendence and control.

What are the remedies best calculated to obviate the present defects? They consist of three,—namely, the conveyance of pure water from ten or twenty miles distance to the metropolis; and to destroy the monopoly which so glaringly exists; or to sink wells, or fountains. The city of Edinburgh was once situated as London now is, but excellent water was soon discovered, and conveyed a distance of ten or twelve miles: and cannot such an example be followed by the most wealthy and populous city in the world? All will admit that such an undertaking would be most readily accomplished. The Thames water might be dispensed with altogether for internal use. Two centuries ago, Sir Hugh Middleton succeeded in bringing pure water from Hertfordshire to the capital, a distance of forty miles. "What is there (says Mr. Wright, in his interesting and valuable Memoir to the commissioners,) that should deter the inhabitants of the richest, largest, most populous city in the world, the seat of more opulent nobility and gentry than is to be found in any other metropolis, from attempting one of those mighty efforts which fix the character of a country, and elevate it in the scale of nations, to remove from that city a *national disgrace*." The day must soon arrive when these suggestions will be attended to, and an object of such vital importance to the health and lives of nearly a million of inhabitants carried into effect. New pipes and tubes could be laid down, for the sole purpose of conveying pure water, and perfectly disconnected with sewers of any kind; and the present supply should be improved, and be continued for the inferior domestic uses.

### METEOROLOGICAL JOURNAL,

*From May 20th, to June 20th,* 1828.

By Messrs. HARRIS and Co. Mathematical Instrument Makers, 50, High Holborn.

| May | Rain gauge. | Moon. | Thermom. | | | Barometer. | | De Luc's Hygrom. | | Winds. | | Atmospheric Variations. | | |
|---|---|---|---|---|---|---|---|---|---|---|---|---|---|---|
| | | | 9 A.M. | MAX. | MIN. | 9 A.M. | 10 P.M. | 9 A.M. | 10 P.M. | 9 A.M. | 10 P.M. | 9 *a.m.* | 2 *p.m.* | 10 *p.m.* |
| 20 | | | 55 | 66 | 51 | 29.67 | 29.60 | 90 | 94 | E | ENE | Fair | Fine | Cloudy |
| 21 | .57 | ☽ | 54 | 63 | 50 | 29.46 | 29.45 | 97 | 98 | ESE | ESE | Rain | Cloudy | —— |
| 22 | | | 51 | 60 | 49 | 29.44 | 29.46 | 98 | 98 | ESE | ESE | —— | —— | —— |
| 23 | | | 54 | 63 | 47 | 29.47 | 29.48 | 92 | 98 | ENE | E | Fair | —— | Rain |
| 24 | .15 | | 54 | 68 | 57 | 29.35 | 29.32 | 98 | 95 | SW | WSW | Cloudy | Rain | Cloudy |
| 25 | | | 59 | 63 | 53 | 29.65 | 29.69 | 97 | 95 | W | WSW | Fair | Fine | Fine |
| 26 | .20 | | 62 | 69 | 55 | 29.57 | 29.46 | 95 | 90 | SW | S | —— | Rain | Cloudy |
| 27 | | | 67 | 70 | 52 | 29.46 | 29.42 | 78 | 86 | SSW | SW | —— | —— | —— |
| 28 | | | 58 | 63 | 57 | 29.47 | 29.53 | 78 | 92 | W | WSW | Cloudy | Fair | —— |
| 29 | .18 | ○ | 63 | 70 | 57 | 29.56 | 29.65 | 80 | 90 | WSW | WSW | Fair | —— | Rain |
| 30 | | | 65 | 71 | 56 | 29.85 | 29.82 | 79 | 90 | WNW | W | —— | —— | Fair |
| 31 | | | 64 | 69 | 55 | 29.71 | 29.78 | 79 | 90 | WNW | WNW | —— | —— | —— |
| June | | | | | | | | | | | | | | |
| 1 | | | 62 | 69 | 47 | 29.91 | 29.97 | 79 | 79 | WNW | W | —— | Fine | —— |
| 2 | | | 53 | 70 | 53 | 29.94 | 29.83 | 79 | 79 | W | WNW | —— | Show'ry | —— |
| 3 | | | 60 | 67 | 55 | 29.85 | 29.76 | 78 | 83 | NW | W | —— | Fair | Cloudy |
| 4 | 100 | ☾ | 58 | 68 | 52 | 29.39 | 29.35 | 94 | 96 | W | W | Rain | —— | —— |
| 5 | | | 57 | 63 | 53 | 29.34 | 29.41 | 96 | 88 | W var. | W | Fair | —— | —— |
| 6 | . 0 | | 56 | 64 | 51 | 29.57 | 29.67 | 77 | 78 | NW | NW | Rain | —— | —— |
| 7 | | | 58 | 63 | 53 | 29.93 | 30.01 | 78 | 78 | NW | NW | Fair | —— | Fair |
| 8 | | | 57 | 66 | 54 | 30.04 | 30.06 | 75 | 78 | NW | NW | —— | —— | —— |
| 9 | | | 58 | 70 | 55 | 30.08 | 30.09 | 78 | 78 | NW | NNW | —— | Fine | —— |
| 10 | | | 63 | 69 | 60 | 30.12 | 30.13 | 78 | 78 | NNW | NNW | —— | —— | —— |
| 11 | | | 65 | 73 | 61 | 30.10 | 30.08 | 76 | 77 | NNW | NNW | —— | —— | —— |
| 12 | | ● | 63 | 65 | 56 | 30.10 | 30.10 | 75 | 75 | NNW | SSE | —— | Fair | —— |
| 13 | | | 62 | 71 | 54 | 30.11 | 30.10 | 75 | 75 | SSE | SSE | —— | Fine | —— |
| 14 | | | 63 | 71 | 56 | 30.11 | 30.07 | 75 | 78 | SE | SSE | —— | —— | —— |
| 15 | | | 64 | 71 | 61 | 30.00 | 29.93 | 92 | 92 | S var. | SE | Rain | Fair | —— |
| 16 | | | 67 | 69 | 59 | 29.83 | 29.79 | 90 | 98 | SSE | ENE | —— | —— | —— |
| 17 | | | 64 | 73 | 60 | 29.41 | 29.40 | 84 | 97 | SW | SE | Fair | —— | Fine |
| 18 | | | 63 | 70 | 60 | 29.30 | 29.60 | 96 | 84 | E | W | Rain | Cloudy | —— |
| 19 | | | 63 | 72 | 60 | 29.80 | 29.82 | 92 | 84 | WSW | SW | Cloudy | —— | Cloudy |

The quantity of Rain fallen in the month of May, was 1 inch and 23.100ths.

